# Impact of feed, light and access to manipulable material on tail biting in pigs with intact tails

**DOI:** 10.1186/s13028-023-00716-8

**Published:** 2024-01-09

**Authors:** Per Wallgren, Magnus Johansson, Torun Wallgren, Zeljko Susic, Kerstin Sigfridson, Sven-Erik Johansson

**Affiliations:** 1 National Veterinary Institute, SVA, 751 89 Uppsala, Sweden; 2 Department of Clinical Sciences, Swedish University of Agricultural Sciences, Box 7054, 750 07 Uppsala, Sweden; 3 Nibble Farming, 725 95 Tillberga, Västerås Sweden; 4 Department of Animal Environment and Health, Swedish University of Agricultural Sciences, Box 7068, 750 07 Uppsala, Sweden; 5 Lantmännen Farming, Box 407, 751 06 Uppsala, Sweden; 6 Lantmännen Farming, Box 1784, 205 03 Malmö, Sweden

**Keywords:** Daylength, Hay silage, Illumination

## Abstract

**Background:**

Tail biting (TB) is a welfare issue with economic consequences due to infections and ill-thrift. This study aimed to reduce tail injuries in a high-performing non-tail-docking pig herd.

**Results:**

During eleven years preceding the trial, the annual incidence of tail injuries registered at slaughter in pigs from the herd increased from 3% (equivalent to the national mean) to 10%. It was positively correlated to a high weight gain and negatively correlated to daylight length.

The overall incidence of tail injuries during the four years preceding the trial was 9.2% with significant differences between four identically structured buildings for fatteners (I < II < III < IV).

The feed was enriched with amino acids, minerals and fibres. The buildings used different illumination strategies, I: standard fluorescent tubes with an invisible flickering light of 30–40% for 14 h daily, II: non-flickering led light for 14 h daily, III (control) and IV: standard fluorescent tubes for 2 h daily. IV had free access to manipulable material (hay-silage), while I–III was offered 100–200 g daily.

During the adaptation period (6 months), the incidence of tail injuries decreased significantly in all buildings to a mean of 5.4%. The largest decrease (from 11.4 to 4.3%) was obtained in IV.

During the trial period (12 months), the mean incidence of tail injuries decreased in all groups to a mean of 3.0%. There were no differences in treatment incidences of individual pigs due to TB between groups, but the use of enriched pellets due to TB in pens was lowest in II. The low incidence of tail injuries was retained during the post-trial period (6 months) when all buildings used artificial illumination for two hours per day.

**Conclusions:**

The incidence of TB in fast growing non-tail-docked pigs in the herd was successfully reduced by supplementing the feed with amino acids, minerals, vitamins and fibres. Additional manipulable material accelerated that process and non-flickering illumination may have had an impact in preventing TB.

The results obtained do not support the need for tail-docking of pigs, provided that the needs of the pigs in terms of feed ingredients, stocking density and access to manipulable materials are fulfilled.

## Background

The tail of mammals is poorly drained by the lymphatic system [1] and the defence towards local caudal infections is therefore not optimal. Thus, the lymphatic system of the porcine tail is less developed than in other body parts and this may contribute to a less efficient immune response in case of tail infection. A common type of infected wounds at the caudal part of the pig are due to tail biting (TB) from where infections risk to spread to other parts of the body. This explains why abscesses, arthritis and total condemnations at slaughter more often are identified in tail bitten pigs than in non-bitten pigs [2–5]. TB causes a wound that is usually infected by oral, fecal and environmental bacteria and the infected wound may be profound involving the vertebrae and may be associated with lymphatic or hematogenous spread which induce a strong inflammatory immune response [6] and therefore require antibiotic treatment to avoid complications such as pyemia [7]. TB is a sign of reduced welfare of the biting pig [8], but also causes pain and ill-thrift [9] as well as decreased weight gain [10–12] in the bitten pigs. Consequently, apart from welfare aspects, there is also an economic reason to prevent TB. A common way to prevent TB is docking tails of piglets, and according to a survey from 24 countries 77% of the pigs reared for slaughter (Median = 95%) were tail docked [13]. That report also concluded that the veterinary profession has a significant role to play in raising awareness and knowledge regarding the benefit of pig health and welfare.

Access to manipulable materials such as straw reduces unwanted behaviours such as TB through enabling exploratory behaviour [9]. Although the lack of straw has been identified as a main risk for the development of TB [14], TB is of multifactorial origin and the triggering factor is often difficult to specify [15, 16]. There are different backgrounds to TB, covering everything from a single pig biting the rest of the pigs in a pen where the solution is to remove the offending pig, to situations where all pigs bite and become bitten [17]; a situation that may be associated with the composition of the feed [10, 18], competition [19] or ventilation errors [20]. It has also been observed that especially young fatteners explore behaviours such as belly nosing and manipulating the tail of other pigs [21] and due to the natural attraction pigs have to blood, TB may arise if bleeding accidently evolve [22].

This study aimed to reduce the prevalence of tail injuries registered at slaughter (presumably caused by TB) in a high-performing non-tail-docking pig herd with increasing incidences of tail injuries registered at slaughter. The methods used included a) supplementing the feed with amino acids, minerals, vitamins and fibres to all pigs; b) different illumination strategies, with the aim to validate the influence of duration and quality of light (including daylength) and; c) different access to manipulable material in terms of hay silage.

## Methods

### Herd

**Fig. 1:**
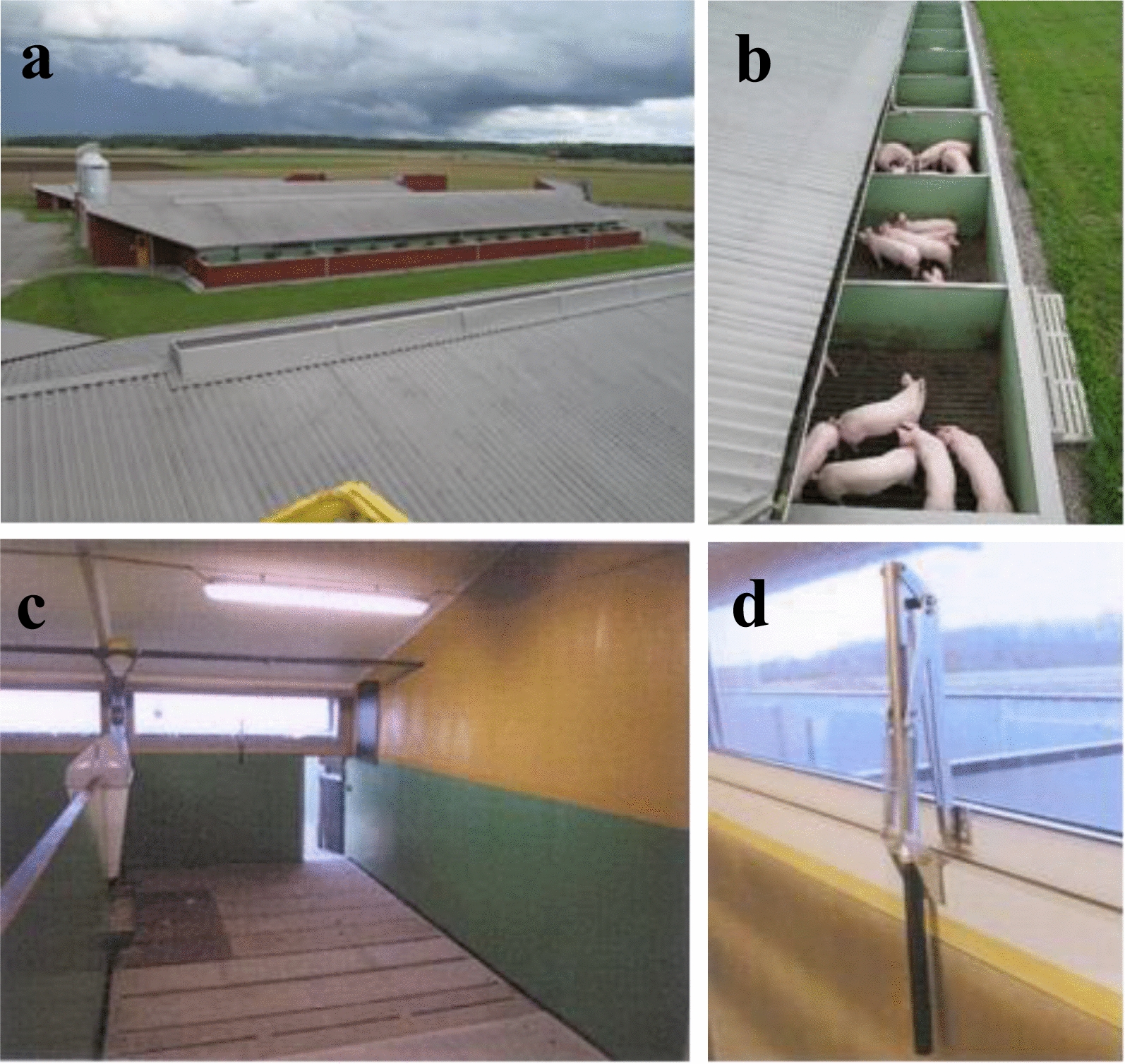
**a** The four buildings for fatteners were in a slope to the fields, 15 m apart from each other with the aim to reduce transfer of microbes between the buildings. To improve the biosecurity, an entrance for staff with possibilities to change shoes and wash hands that also stored hay silage was built on the gable to the left of each building and a space for delivering market weight pigs to slaughter was built on the gable to the right of each building. **b** Pigs had access to an outdoor area that also was used for dunging. **c** Each unit with two pens that housed 20 pigs each had ventilation and manure systems that were separated from the other units. Each pen had two automatic feeders where four pigs could eat simultaneously and the door under the windows lead to the outdoor facility. **d** The mechanical ventilation was regulated through the windows by a bimetallic window opener. Courtesy of PerArne Mattsson

The study scrutinised tail injuries presumably caused by TB in an integrated conventional high health herd that reared non-tail-docked pigs for slaughter in a rearing system with improved animal welfare (Fig. 1). Fatteners had improved opportunities for exploratory behaviours and access to an outdoor space [23, 24].

The herd had 230 sows and employed an age segregated all in-all out-production system from birth to slaughter, and all facilities housing growing pigs were washed and disinfected between batches. Every 19th day, 25 Yorkshire-Landrace sows mated with Duroc gave birth to piglets in a farrowing unit. Male piglets were castrated at the age of three days, and the non-tail-docked piglets were weaned and transferred to a weaning unit with large pens for up to 100 piglets per pen at a mean age of 32 days.

At the mean age of 65 days, piglets with a mean weight of 26.3 kg were transferred to a fattening unit. By 2010, four identically structured buildings with fattening pens aimed to improve the well-being of the fatteners were inaugurated [23, 24]. The four buildings for fatteners were in a slope to the fields, 15 m apart from each other with the aim to reduce transfer of microbes between the buildings. To improve biosecurity, an entrance for staff with possibilities to change shoes and wash hands that also stored hay silage was built on one gable, and a space for delivering market weight pigs to slaughter was built on the other gable of each building (Fig. 1a). Each pen had access to a dunging area located outdoors (Fig. 1b).

The pens were larger than common fattening pens. They were sized 19.6 m^2^ and included a lying area and an eating area of 13.9 m^2^ indoors (Fig. 1c) as well as a dunging area of 5.7 m^2^ located outdoors. The indoor area of each pen had windows spaced 1.37 m^2^, corresponding to 0.1 m^2^ window area per m^2^ of the indoor floor area (Fig. 1d). Due to the presence of windows and outdoor access, artificial illumination with fluorescent tubes was limited to care-taking time of the pigs, around two hours per day.

Each building housed 15 units (rooms) with an individual mechanical ventilation system regulated through the windows by a bimetallic window opener (Fig. 1d) and each unit (room) had two pens sized 19.6 m^2^. Each pen initially housed 20 pigs (selected from the large pens of the weaning unit), corresponding to 0.98 m^2^ per pig, with free access to dry feed from four positions (5 pigs per position). With the aim to stimulate explorative behaviour and mimic feed search, pigs had to push/pull levers to release feed from the automatics. The pigs also had free access to water from three nipples (6.7 pigs per nipple). To harmonise with the animal welfare law of Sweden [25], the largest 1–2 pigs were slaughtered when the mean weight of the pigs in a pen reached 100 kg. The area demanded per pig in Sweden is 0.40 m^2^ per pig at 30 kg weight and 0.94 m^2^ per pig at 100 kg weight. Corresponding figures within EU is 0.30 m^2^ at 30 kg weight and 0.65 m^2^ at 100 kg weight [26].

### Before initiating the trial

The fattening pigs were offered a dry meal feed *ad lib* from totally four eating positions per pen as described above. Data on composition of feed recipes are unpatented trade secrets and therefore not publicly available. Here, the feed recipes used were optimised by the leading feeding company of Sweden (Lantmännen, Malmö, Sweden), and the feed consequently corresponded to the mean standard for feed to fatteners in the country. The diets were mixed on farm using barley, wheat, outs, faba beans, field peas rape seed meal, soya bean meal linseed expeller, dry fat and a special adapted premix according to the standard Swedish recommendations. Over time the on-farm recipes were adapted to the quality and availability of the raw materials (crops) produced on the farm. The pigs were also offered hay silage per pig daily during the entire rearing period, 100–200 g per day (depending on the density of the silage).

On average, pigs were slaughtered 105 days after arrival to the fattening units at a mean weight of around 130 kg. The live weight at slaughter was calculated as slaughter weight (kg) * 1.34. The daily weight gain (DWG) during the fattening period was calculated as follows: [kg live weight at slaughter − 26.3 kg (estimated weight on arrival to fattening unit)]/105 days (estimated rearing period at the fattening unit)]. All pigs were slaughtered at one abattoir (Skövde Slakteri Ltd, Skövde, Sweden). The DWG, the meat percentage of the carcasses and the incidence of tail injuries registered at slaughter registered were calculated from abattoir data. Tail injuries (presumably TB) was recorded at slaughter according to instructions from the Swedish Food Agency [27] demanding registration of all visible tail injuries, *i.e.*including ulcers, visible wounds, mechanical lesions, and short tails with as well as without visible wounds in the skin. Treatment of pigs and mortality during the growing period were registered at the herd.

**Fig. 2:**
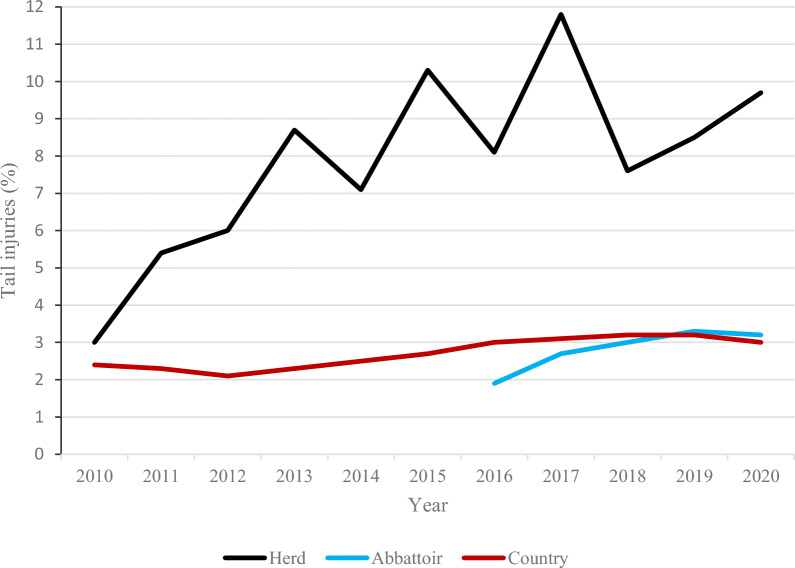
The absolute incidence of tail injuries (presumably caused by tail biting) recorded at slaughter in the herd before the trial (black line). There was a positive correlation (P < 0.0001, generalised linear model test, least square means comparisons) between daily weight gain and tail injuries. The figure also shows the mean incidence of tail injuries in the abattoir where the pigs were slaughtered (blue line) and in Sweden in general (red line) over the years

Due to the high DWG during the fattening period and the increasing incidence of tail injuries registered at slaughter (Fig. 2), adjustments of the feed took place several times from 2015 and onwards, mainly focusing on minerals and amino acids but also on increased levels of fibres (see below) with the aim to reduce the TB incidence.

Further, the overall monthly incidence of tail injuries registered at slaughter was analysed and compared with the daylength for the total period of eleven years (2010–2020). The daylength (sunrise to sunset) at the location of the herd was defined by the Swedish Meteorological and Hydrological Institute (SMHI, Norrköping, Sweden).

The incidence of tail injuries registered at slaughter (presumably caused by TB) and the DWG during the last four years before initiating the trial (2017–2020) were documented in detail for each building (each building had a unique identity at the abattoir). These four years are referred to as the comparison period.

### Trial design

The trial was initiated on the first of January 2021. The four buildings for fatteners were randomly allotted into four experimental groups. As each building had 15 separate units (rooms) that housed 40 fatteners, pigs were distributed to the different buildings at allocation without interfering with the all in-all out concept at room level. The following artificial illuminations were implemented from 1 January 2021:

Group I (Building 1, closest to the central units with sows and piglets): Ordinary artificial illumination with fluorescent tubes from 06.00 to 20.00; The invisible flickering in standard fluorescent tubes generally vary from 30 to 40% and they also may induce electromagnetic interference (EMI), especially at the interval from 104 to 1012 Hertz which may impair electric equipment and mammals negatively [28]. The aim was to validate the impact of an extended illumination with standard fluorescent tubes.

Group II (Building 2, second closest to the central units): Artificial illumination free from invisible flickering (< 0.3%) and not generating any EMI (Uni-light IP65T8B, Uni-light Led Ltd, Stockholm, Sweden) from 06.00 to 20.00. The length of the illumination intended to be long enough to decrease the melatonin production in the pigs during daytime with the aim to create harmony in the pens [29].

Group III (Building 3, second closest to the open fields): Ordinary artificial illumination with fluorescent tubes during caretaking, approximately 2 × 1 h per day. The aim was to create a control group with the same lighting conditions as before the trial.

Group IV (Building 4, closest to the open fields): Ordinary artificial illumination as in Group III, *i.e.,* two hours per day during caretaking. In this building, the pigs had free access to hay silage, offered in racks (Width 70 cm, Depth 28.5 cm Height 47 cm; PB 127, Siltbergs smide, Visby, Sweden) mounted on the walls with the lowest point 40 cm above the floor level. The aim was to validate the impact of increased access to manipulable material.

By September 2020, a new recipe of the premix for fatteners was implemented (405,651 Delta Mix P6209, Lantmännen, Malmö, Sweden), in which the levels of the amino acids lysine, methionine, threonine and tryptophan were increased by 10% over the Swedish standard/Company norm; calcium, phosphorus, sodium, chlorine, magnesium were 20% above that norm. The levels of vitamin A, B_1_, B_2_, B_3_, B_5_, B_6_ and B_12_ was increased by 10–20%, vitamin D was increased with 40% and vitamin E was increased with 80% compared to the norm. The premix was mixed with soya bean meal, rape seed meal and dry fat (Lipitec ® piggy; NLM Vantinge A/S, Ringe, Denmark) and cereals produced on the farm (barley, wheat, oats, peas, and Swedish faba beans (the minuta group of *Vicia faba*) into three different feeds with 12.4–12.6 MJ metabolisable energy (ME) per kg that was offered *ad lib* to all fatteners. The phase I (from 63 to 90 days of age), II (from 91 to 120 days of age) and III (from 121 days of age) feed included 18.0%, 17.4% and 14.3% protein, respectively.

From the 1st of July 2021, large (23 mm) and solid fodder pellets (406,889 Time Out; Lantmännen, hereafter referred to as Time Out-pellets) was offered on the floor to pens where the animal caretakers perceived apprehensiveness or observed TB in more than two pigs. The Time Out-pellets were a spin-of product to the premix 405,651 Delta Mix P6209 that was initiated in September 2020 and is described above. The aim was to prevent TB by occupying the pigs with the large and solid pellets that also included minerals and fibres. The daily dose was 30–35 g per pig for 10–14 days.

### Classifying of results obtained

The rearing period for fatteners was 105 days. Consequently, pigs slaughtered from 1 January to 15 April 2021 had not spent their entire fattening period illuminated as described above. Nor were Time Out-pellets, with an expected immediate effect (if any) used prior to 1 July. For these reasons, the period from 1 January to 30 June 2021 was separately validated and is referred to as the adaptation period.

The trial period was from 1 July 2021 to 30 June 2022. During this period all pigs slaughtered had experienced the illumination programs described above during the entire fattening period and the Time Out-pellets were offered to all pens where the staff perceived apprehensiveness of pigs or TB was observed in more than two pigs.

During both periods, the number of pigs slaughtered and the number of pigs with tail injuries at slaughter (presumably caused by TB) was registered per building, and the incidence of tail injuries at slaughter per building was calculated.

Clinical observations were recorded in terms of number of individual fatteners treated with antimicrobials for treatment of tail bites during the trial period, as well as the number of pens offered Time Out-pellets for 10 to 14 consecutive days when apprehensiveness /TB was perceived/observed in more than two pigs.

The illuminance was quantified as LUX (1 lm per m^2^), using a luxmeter (48,882, Mini Light Meters 48,882, UNI-Trend Technology, Songshan, China) 50 cm above floor level (pig height) at noon in five randomly selected pens in all buildings following cleaning and disinfection.

The weight gain during the fattening period was calculated as described above, *i.e.* (estimated live weight at slaughter–estimated weight on arrival) /estimated rearing period, corresponding to [(kg slaughter weight * 1.34) − 26.3 kg]/105 days.

### The post-trial period

The incidence of tail injuries registered at slaughter and presumably caused by TB was also registered for a period of 6 months after ending the trial (1 July to 31 December 2022). All pigs consumed the same food as during the trial, but Groups I and II had returned to an illumination period of around two h per day during caretaking. During this period, pigs in all buildings (including Group IV) received 100–200 g hay silage per pig and day.

### Statistics

With the aim to create a background (control) for the evaluation of the trial, and to compensate for any differences in performance between the buildings, the incidence of tail injuries registered at slaughter and the DWG was documented in detail for each building during the comparison period (2017–2020). These four years are referred to as the comparison period. Thus, each building was its own control when evaluating the trial. In addition, the buildings were compared with each other.

The incidence of tail injuries registered at slaughter and presumably caused by TB, as well as treatments due to TB are presented as total incidence, *i.e*., number of affected pigs divided with the number of pigs that were slaughtered. Incidences of tail injuries as well as individual treatments with antimicrobials and pen treatments with Time Out-pellets during the trial were compared using χ^2^-tests between buildings (groups) during study periods and within buildings between different study periods.

When mean values were calculated, they are presented as mean values ± standard deviations. Differences in weight gains and meat percent of carcasses with normal distributions were analysed with student’s t-tests.

The difference between incidence of TB or DWG in different periods were investigated with the non-parametric Kruskal–Wallis test. The difference in DWG over years was also investigated through a generalised linear model with least square means (LSM) adjusted for multiple comparisons with Tukey Kramer.

To investigate the effect of daylength on tail injuries for the whole herd before the study was initiated (2010–2020), a fixed effects general linear model was created with the percentage of TB per month as outcome and year, daylength and DWG as fixed effects. The final model was created through backwards elimination.

To investigate the effect of daylength on tail injuries during the trial period a fixed effects model was created with the percentage of TB per month as outcome and daylength and stable (treatment) as fixed effects. In order to get the residuals normally distributed to fit the model, the data was log transformed.

When investigating the effect of daylength, referred to as a generalised linear model test, LSM were adjusted for multiple comparisons through Tukey–Kramer for multiple comparisons. The statistical analyses were performed with SAS 9.4. Proc GLM was used to estimate the effect of different variables on TB. Treatment, year and daylength was included in all the models. Treatment was only relevant and included during the adaptation and trial periods. There were no significant interactions between the variables. For significant variables, differences were investigated further through LSM.

## Results

### Results obtained before initiating the trial

During the first three years using the new fattening enterprises (2010–2012; 18,070 pigs slaughtered), the mean DWG was 958 ± 8 g per day, which increased significantly (P < 0.0001, Kruskal–Wallis test) to 997 ± 45 g per day during the subsequent eight years (2013–2020; 42,234 pigs slaughtered). Also the incidence of tail injuries registered at slaughter and presumably caused by TB increased significantly (P < 0.0001, Kruskal–Wallis test), from 4.8 ± 1.6% during 2010–2012 to 9.0 ± 1.5% during 2013–2020 (Fig. 2). The annual mean mortality rate in the fattening enterprises were 3.2 ± 1.2%.

During the eleven years that preceded the trial (2010–2020), the overall mean incidence of tail injuries registered at slaughter was 7.7 ± 4.2% (60,304 pigs slaughtered). The incidence of tail injuries was significantly (P < 0.0001, generalised linear model, LSM comparisons) affected by year and also by weight gain, *i.e*. the higher weight gain, the higher incidence of tail injuries (Fig. 2).

**Fig. 3:**
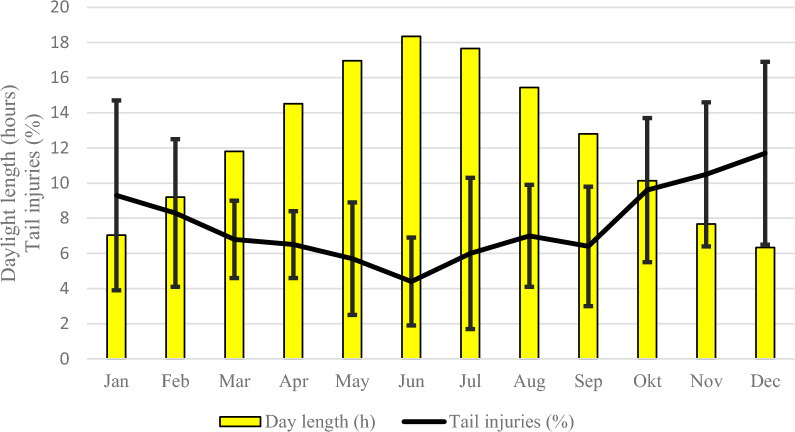
The correlation between daylength (sunrise to sunset) and tail injuries registered at slaughter before the trial (2010–2020). There was a negative correlation (P < 0.0001, generalised linear model test, least square means comparisons) between daylength and tail injuries

The incidence of tail injuries presumably caused by TB was also significantly (P < 0.0001, generalised linear model, LSM comparisons) influenced by daylength in a negative way, *i.e.* the shorter days the higher incidence of tail injuries (Fig. 3). December with the shortest daylength had significantly higher incidences of tail injuries than each of the months from March to September (P < 0.05 to < 0.0001). June with the longest daylength had significantly lower incidences of tail injuries during each month from October to January (P < 0.05 to < 0.0001).

### Results obtained during the comparison period

During the four years that preceded the trial (2017–2020), the overall incidence of tail injuries registered at slaughter was 9.2% (1572 out of 17,037 pigs). However, as seen in Table 1, the incidence of tail injuries differed significantly (P < 0.05, χ^2^-test) between all buildings. According to the generalised linear model, the percentage of TB was significantly affected by year (P < 0.0001), daylength (P = 0.0108) and stable (P = 0.0134). In contrast, the meat percentage at slaughter and the daily weight gain (DWG) of the pigs during the fattening period was similar. The annual mean mortality rate in the fattening enterprises were 3.0 ± 1.4%.

**Table 1:** Tail injuries registered at slaughter (presumably caused by tail biting) and productivity in the four buildings during the comparison period (2017–2020)

Building	I	II	III	IV
Slaughtered (n)	4540	4208	4042	4247
With tail injury (n)	299	368	402	503
With tail injury (%)	6.6^ABC^	8.7^AD^	9.9^BE^	11.8^CDE^
DWG (fattening period) (g/day)	1024 ± 90	1019 ± 74	1030 ± 145	1015 ± 75
Meat% at slaughter (%)	58.6 ± 1.1	58.6 ± 1.3	58.7 ± 1.4	58.7 ± 1.1

Pigs entered the fattening facilities at a mean weight of 26.3 kg and were slaughtered at a mean weight of around 130 kg

Within lines, columns with identical letters differ significantly [P < 0.01 (A and E); P < 0.001 (B, C and D)]

### Light intensity during the adaptation period and during the trial

When measured 50 cm above floor in the middle of a pen at noon, the light intensity corresponded to 310 LUX when the standard artificial illumination with fluorescent tubes were lightened (Group I), and to 180 LUX when the lights were off (Groups III and IV). When the non-flickering led tubes were lightened, the light intensity corresponded to 245 LUX (Group II). When measured outdoors, the light intensity in sunshine was 4200 LUX.

### Results obtained during the adaptation period

**Fig. 4:**
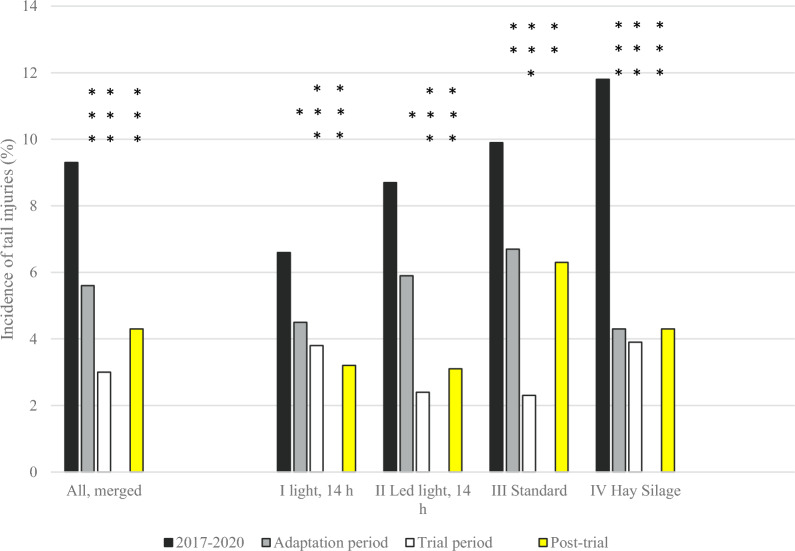
The exact incidences of tail injuries (number of tail injuries/number of slaughtered) for the four individual buildings, as well as merged for the whole herd. The comparison period before the trial corresponded to 2017–2020.The adaptation period was from January to June 2021. The trial period was from July 2021 to June 2022. The post-trial period was from July to December 2022. The stars in the figure represent significant differences compared to the comparison period within unit (χ^2^-tests; *P < 0.05; **P < 0.01; ***P < 0.001). At herd level, the incidence of tail injuries during 2017–2020 was higher (P < 0.01 to < 0.0001, Kruskal–Wallis test) than during the other periods. The overall level of tail injuries was also higher (P < 0.01) during the adaptation period compared to the trial period

During the adaptation period (1 January to 30 June in 2021) the overall incidence of tail injuries presumably caused by TB decreased significantly (P < 0.01, χ^2^-test) from 9.2 to 5.4% (137 out of 2556). As seen in Fig. 4, the incidence of tail injuries decreased with 32% in Groups I, II and III (from 6.6 to 4.5% in Group I (P < 0.05, χ^2^-test); from 8.7% to 5.9% in Group II (P < 0.05, χ^2^-test); from 9.9 to 6.7% in Group III (P < 0.01, χ^2^-test), and with 64% in Group IV (from 11.8% to 4.3%% (P < 0.001, χ^2^-test). During this period there were no significant (P > 0.05, χ^2^-tests) differences in incidence of tail injuries registered at slaughter between groups/buildings (Table 2). The mean mortality rate in the fattening enterprises was 2.3%, which was significantly (P < 0.001, χ^2^-test) lower than during the comparison period.

**Table 2:** Tail injuries registered at slaughter (presumably caused by tail biting) in the four buildings during the adaptation period of 6 months (January to June 2021)

Building/Group	I Light 14 h	II Led light 14 h	III Standard	IV Hay silage
Slaughtered (n)	624	608	689	635
With tail injury (n)	28	36	46	27
With tail injury (%)	4.5	5.9	6.7	4.3

There were no significant differences between any groups (P > 0.05, χ2-test)

### Results obtained during the trial period

During the trial period of one year (1 July 2021 to 30 June 2022), the overall incidence of tail injuries registered at slaughter and presumably caused by TB had decreased with 67%, from a mean of 9.2% during the comparison period to a mean of 3.0% when all buildings were merged (162 out of 5345), which differed significantly (P < 0.001, χ^2^-test) from the comparison period (2017–2020; 1572 out of 17,037 pigs = 9.2%; Fig. 4). The mean mortality rate in the fattening enterprises was 1.9%, which was significantly (P < 0.001, χ^2^-test) lower than during the comparison period—but not lower than during the adaption period (P > 0.05, χ^2^-test).

The incidences of tail injuries registered at slaughter were significantly (P < 0.001, χ^2^-test) lower within all groups than during the comparison period (Fig. 4). However, the decrease within building was lower in Group I (42%, from 6.6 to 3.8%) than in the other buildings where the decrease ranged from 69 to 77% (Group II: 72%, from 8.7 to 2.4%; Group III: 77% from 9.9 to 2.3%; Group IV: 69%, from 11.8 to 3.6%). Therefore, the incidence of tail injuries during the trial was significantly (P < 0.05, χ^2^-test) higher in Group I than in Groups II and III (Table 3).

**Table 3:** Tail injuries in the four buildings registered at slaughter during the trial that was carried out for one year (July 2021 to June 2022)

Building/Group	I Light 14 h	II Led light 14 h	III Standard	IV Hay silage
Slaughtered (n)	1331	1295	1418	1301
With tail injury (n)	51	31	33	47
With tail injury (%)	3.8^A^	2.4^BC^	2.3^B^	3.6^AC^
DWG (fattening period) (g/ day)	976 ± 89	987 ± 63	960 ± 89	950 ± 92
Meat% at slaughter (%)	58.1 ± 1.1	58.2 ± 1.3	58.4 ± 1.0	58.3 ± 0.8

Pigs entered the fattening facilities at a mean weight of 26.3 kg and were slaughtered at a mean weight of around 130 kg

Within lines, columns with different letters differ significantly (P < 0.05, χ^2^-test/t tests)

As also seen in Table 3, there were no significant differences in DWG between the different groups/buildings (P > 0.05, Kruskal–Wallis test) when the standard formula for estimating the DWG was used.

**Fig. 5:**
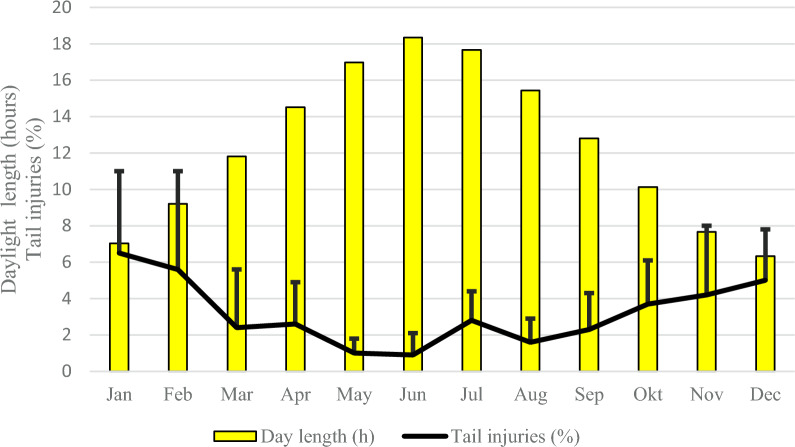
The correlation between daylength (sunrise to sunset) and tail injuries registered at slaughter during the trial when the mean incidence of tail injuries was 3.0%

The overall relationship between daylight and tail injuries during the trial that lasted for one year is shown in Fig. 5. No significant (P > 0.05, generalised linear model, LSM comparisons) effect of daylength or stable (treatment) on tail injuries were found during the trial period. It should however be noted that the trial was carried out for one year and thereby included fewer observation points per month than during the eleven years that preceded the trial (Fig. 2).

As seen in Table 4, the merged incidence of individual treatments due to TB or perceived apprehensiveness of pigs by the staff ranged from 1.1 to 2.0% and there were no significant (P > 0.05, χ^2^-test) differences between the different groups/buildings. In contrast, the number of pens offered Time Out-pellets for 10–14 days was significantly (χ^2^-test) lower in Group II than in the other groups/buildings, (P < 0.05 compared to Group I and P < 0.001 compared to Group III and IV).

**Table 4:** Merged treatments of individual pigs with antibiotics due to unsettlement or due to tail biting in the four buildings during the trial

Building/Group	I Light 14 h	II Led light 14 h	III Standard	IV Hay silage
Pigs slaughtered(n)	1331	1295	1418	1301
Individually treated				
Tail bitten or bitten (n)	27	22	16	22
Tail bitten or bitten (%)	2.0	1.7	1,1	1.7
Pens used (n)	67	65	71	66
Pens given Time Out				
Treated (n)	10	2	16	16
Treated (%)	14.9^A^	3.1^B^	23.9^A^	24.2^A^

The table also shows the incidence for spread of Time Out-pellets to pens when unsettlement/tail biting on pen level was observed

Within lines, columns with different letters differ significantly (χ^2^-tests; P < 0.05 II vs I; P < 0.001 II vs III and IV)

### Results obtained during the post-trial period

During the post-trial period of six months (July to December 2022), the overall incidence of tail injuries was 4.3% (n = 121 out of 2960 pigs slaughtered), which was significantly (P < 0.0001, χ^2^-test) lower than during the comparison period (n = 1572 out of 17,037 pigs, 9.2%). The incidences were 3.2% in Group I, 3.1% in Group II, 6.3% in Group III and 4.3% in Group IV (Fig. 5). The mean mortality rate in the fattening enterprises was 2.3%, which was significantly (P < 0.001, χ^2^-test) lower than during the comparison period—but not lower than during the adaption period and the trial period (P > 0.05, χ^2^-test).

## Discussion

Despite a potentially increased welfare with larger fattening pens, outdoor access and a low use of antimicrobials [23, 24], the high incidence of tail injuries registered at slaughter and presumably caused by TB had remained high before the trial. Thus, it was an exemption that tail injuries decreased to the national mean level of around 3% (Fig. 2) in all four groups/buildings during the trial (Fig. 5), and that a low incidence of tail injuries was maintained during the post-trial period (the somewhat higher incidence post-trial was caused by individual batches with high levels of tail injuries in Groups III and IV, data not shown). In addition, the mortality during the fattening period was decreased with 33% (from 3 to 2%).

As the incidence of tail injuries before initiating the trial clearly was correlated to daylight (Fig. 3) it was important to set up the trial for a full year to avoid bias by any seasonal effects. Also, we had to consider that the fattening buildings were populated with different ages of fatteners when the illumination strategies were implemented as of 1 January 2021. Therefore, we used the first six months of 2021 as an adaptation period that also comprised the full variation in daylength over the year. Thereafter the trial period of a full year followed (from July 2021 to June 2022). Similarly, the post-trial period of six months was important to document a persistency of the decreased incidence of tail injuries after going back to the normal strategy regarding illumination and access to haylage.

Despite identical standard operative protocols regarding care taking, the incidence of tail injuries differed between the identically structured buildings during the comparison period. It was notable that the incidences of tail injuries increased with proximity to the open fields. Non-optimised feed may result in misbehaviour [18] that in turn may have been strengthened by an increased exposure to wind and weather, but we could not prove that. Consequently, each building was made its own control. However, the buildings were also compared with each other.

The incidence of tail injuries presumably caused by TB was significantly reduced in all groups (regardless of treatment) already during the adaptation period. Especially in Group IV with free access hay silage, which underscored the importance of sufficient amounts of manipulating material that previously have been concluded by others [9, 12, 16]. However, initially pigs had difficulties to benefit from the racks with hay silage situated on the pen wall 40 cm above floor level (results not shown). As difficulties for young fatteners to capitalise filaments available from above also have been observed earlier [30] extra hay silage was therefore also offered on the floor. Possibly the efficacy of racks may be increased by incusing piglets to stimuli above floor level through a more stimulating environment, as shown for chicken and mice [31, 32].

As stated, the results obtained during the adaption period clearly indicated a potential of expanded amounts of manipulable materials to prevent TB, as also previously have been concluded by others [9, 12, 16, 32]. However, such materials must represent novelty to the pigs as pigs only will deal with them as long as they consider them as interesting [21, 23, 24]. Therefore, smaller amounts of manipulable materials distributed at shorter time intervals may be desirable, both from a pig and from a manure-handling point of view. Straw has often been kept to a minimum out of fear for problems with the manure handling systems [33], and the average amount of straw per finishing pig in Sweden has been estimated to about 50 g per pig and day [33]. However, it is notable that none of five herds that doubled the amount straw experienced manure handling problems [34], and that an inquiry to Swedish pig farmers stated that problems with manure handling due to straw were rare [33]. Nor were any manure problems observed in this herd where Group IV had free access to hay silage, which apart from including proteins also is tastier than straw and therefore more attractive to the pigs [35–37]. The amount of hay silage used in Group IV probably approached 400 g per pig and day—a ratio that seems to fulfil the exploratory needs of pigs, while straw rations above that do not reduce TB further [38]. Thus, straw and other manipulable materials ought to be looked upon as a potential for the pigs to fulfil natural behaviour rather than as merely bedding material.

During the trial, the overall incidence of tail injuries presumably induced by TB was 67% lower than during the comparison period and the overall incidence corresponded to 3.0%, which in turn corresponded to the national mean of 3.2% tail injuries registered at slaughter in 2018 [39]. The incidence of tail injuries was reduced in all groups, including Group III which remained as a control and implemented an identical illumination strategy as before the trial. Consequently, it appeared that the feed after annual upgrades finally corresponded to the demands of the fast-growing fatteners. The failure of attaining this state earlier may appear odd, not the least since deficient levels of minerals, protein and amino acids (apart from feeder space) risk to induce TB [18]. However, it should be remembered that levels of expensive feed ingredients, such as minerals, amino acids and vitamins, are limited in feed to fatteners that are slaughtered at a young age and feed recipes are adapted to the demands of the mean pigs of a population. Fast-growing pigs therefore risk deficiency of minerals and amino acids [18]. In addition, feed recipes are not protected by patents, so to protect recipes producers only declare the content of energy and protein which is required by law. Thus, optimising feed will be effectuated in small steps, which may delay accomplishment of an optimal feed recipe—as in this case.

Concluding a leading impact of the feed, the relevance of the other parameters investigated may at a first sight appear less valuable. However, it should be remembered that the incidence of tail injuries decreased earlier in Group IV with free access to hay silage than in the other groups, which emphasised the impact of sufficient levels of manipulable material as discussed above.

Nor should the quality of the illumination be neglected. The correlation to daylength observed before the trial clearly showed an effect of the daylight (Fig. 3). The trend was similar, but at a lower level during the trial (Fig. 5). Therefore, it was notable that the incidence of tail injuries in building I, which was lower than in the other buildings during the comparison period, was highest during the trial when these pigs were exposed to the light from standard fluorescent tubes with an invisible flickering of 30–40% for 14 h per day (Group I). The incidence of tail injuries was significantly lower in Group II with an equally long light exposure to non-flickering light, which indicated an impact also of the quality of the light as also have been concluded by others [40, 41]. Nor is sunlight, which was the main illumination in Groups III and IV, flickering.

Sunlight decreases serum levels of melatonin, but it has been shown that blue led-light with a wavelength of 446 to 477 nm induce plasma melatonin suppression in humans [42]. The production of melatonin that induce fatigue is stimulated by darkness, and exposure to blue light ought therefore not exceed 14 h per day due the risk for insomnia induced by a lack of melatonin, which justified the daily length of the illumination in Groups I and II. Properly managed illumination that suppress plasma melatonin in daytime combined with appropriate melatonin production during the night theoretically induce tranquillity and relevant sleeping habits [29]. Seen from this perspective, the lower treatment incidences at pen level with Time Out-pellets due to perceived apprehensiveness of pigs in Group II possibly indicated an increased tranquillity in that building.

Interestingly, an appropriate use of melatonin decreasing light has been correlated to an increased milk production during the early lactation period of cows, but not over time [43] as also strengthened by the fact that melatonin treatment of dairy cattle around drying-off was ineffectual [44]. Accordingly, further studies are needed to evaluate the true impact of melatonin decreasing light on tranquillity and productivity in pigs. Not the least since the treatment incidence of individual pigs due to TB or due to aggressions were not lower in Group II than in the other groups.

Taken together, the present study showed that it is feasible to prevent tail injuries presumably caused by TB in non-tail-docked pigs if the demands for feed ingredients, stocking density and access to manipulable materials are fulfilled. Therefore, it seems odd that tail docking has been practised in many countries for decades. Within EU, tail docking is forbidden by law unless judged as required [26]. However, the possibility to dock tails when judged as needed has led to tail docking within the entire union except in countries where tail docking is prohibited by national laws like Finland and Sweden [11, 13]. Tail docking has been defended by the fact that 1–3% of the pigs in non-docking countries are registered with tail injuries at slaughter [39]. On the other hand, one could interpret the incidence of tail injuries in herds that tail dock to be 100%, and with that perspective, tail docking is not the solution to the problem [45]. Indeed, mutivatile piglet husbandry procedures such as tail docking, teeth clipping and castration by themselves cause behavioural and physiological changes indicative of acute pain that potentially may cause long term negative consequences such as abscesses and formation of neuromas [46]. Additionally, tail docking does not eliminate TB since 3.2–70% of the pigs show signs of TB in tail docked populations [47, 48]. Therefore, there is a growing support for allowing pigs to keep their tails worldwide, primarily for animal welfare reasons [49]. The results of our study, along with previous research that has identified successful strategies for rearing pigs with intact tails, such as providing straw, decreasing stocking density, maintaining high health status, and increasing weaning age [34], further reinforce this argument. Previous findings also suggest that a combination of straw provision and lower stocking density can be as effective in preventing TB as tail docking, as demonstrated under Danish conditions [50].

## Conclusions

During the pre-trial period a high DWG and a short daylength was positively correlated with a higher incidence of tail injuries registered at slaughter.

By supplementing the feed to fast-growing pigs with intact tails with amino acids, minerals, vitamins and fibres, the incidence of TB was successfully reduced. Furthermore, the addition of manipulable materials, such as hay silage, accelerated this process, and non-flickering illumination may have had a preventative impact on TB.

The results obtained strongly suggest that tail docking of pigs is unnecessary, provided that the demands of amino acids, minerals, vitamins and fibres in feed are accommodated.

## Data Availability

Not applicable.

## Abbreviations

DWG: Daily weight gain
DPA: Day post arrival
EMI: Electromagnetic interference
ME: Metabolisable energy
LSM: Least square means
TB: Tail biting
